# Therapeutic effect of eravacycline against carbapenem-resistant hypervirulent *Klebsiella pneumoniae* in mouse models

**DOI:** 10.1128/aac.01237-25

**Published:** 2026-02-23

**Authors:** Ruyi Zhang, Chenchen Zhang, Juan Liu, Xiaoli Kong, Shulei Zhang, Miaosu Chang, Wei Xu, Mei Zhu

**Affiliations:** 1 Department of Clinical Laboratory, The Fourth Affiliated Hospital (Affiliated Chaohu Hospital) of Anhui Medical University), Chaohu, Anhui, People's Republic of China; 2 Department of Blood Transfusion, The First Affiliated Hospital of Anhui Medical University 36639, Hefei, Anhui, People's Republic of China; Shionogi Inc., Florham Park, New Jersey, USA

**Keywords:** CR-hvKP, eravacycline, *in vivo*, therapeutic effect

## Abstract

Carbapenem-resistant hypervirulent *Klebsiella pneumoniae* (CR-hvKP) presents a major challenge for clinical treatment due to its characteristics of drug resistance and hypervirulence. This study aims to investigate the therapeutic effect of a novel tetracycline antibiotic, eravacycline, on CR-hvKP pneumonia. We isolated three CR-hvKP strains from clinical samples and examined their associated phenotypes. Subsequently, we infected mice with strains. The results indicated that the clinical isolates KP78, KP98, and KP100 carried multiple drug resistance genes and virulence genes, exhibiting high virulence levels and significantly resistant to neutrophil-mediated intracellular killing (*P* < 0.01). The body weight of infected mice decreased significantly (*P* < 0.0001). At the same time, the bacteria entered the blood from the lungs and spread to other organs. After the administration of eravacycline, the body weight of the mice began to increase 36 h later. The number of bacterial colonizing in the lungs was reduced (*P*< 0.01), the infiltration of inflammatory cells was reduced, and the interstitial vascular dilatation and congestion were alleviated. Meanwhile, eravacycline significantly suppressed the serum levels of cytokines IL-6, IL-1β, and MCP-1 (*P* < 0.05). Notably, eravacycline suppressed the mRNA expression of STAT1 and p-STAT1 activation in the lung tissue of mice in the treatment group. In conclusion, clinical isolates KP78, KP98, and KP100 caused severe lung injury after invading mouse lung tissues, and eravacycline attenuated the lung injury and inflammatory response induced by CR-hvKP invasion.

## INTRODUCTION

Pneumonia, commonly categorized into hospital-acquired pneumonia (HAP) and community-acquired pneumonia (CAP), is an acute infection that affects the respiratory system. Pneumonia can be caused by infection with a variety of pathogens, including *Klebsiella pneumoniae*, which is associated with high morbidity and mortality (1, 2). After *Klebsiella pneumoniae* enters the host body, the lung is usually the initial site of infection. In lung tissue, *Klebsiella pneumoniae* can survive and damage tissues through substances such as capsular polysaccharide, the synthesis of branched-chain amino acids, and citrate synthase, and even break through the lung barrier to enter the bloodstream, causing bacteremia (3–5). The recent proliferation of drug-resistant *Klebsiella pneumoniae* has significantly increased the mortality among patients with lung infections worldwide (6), under the critical importance of prompt treatment. Unfortunately, despite advances in treatments, *K. pneumoniae* appears to be evolving as well.

Carbapenem-resistant hypervirulent *Klebsiella pneumoniae* (CR-hvKP), a strain that is both highly virulent and resistant to carbapenems, has been widely spread globally (7, 8). Compared with pneumonia caused by classic *Klebsiella pneumoniae* (CKP) with broad-spectrum resistance and hypervirulent *Klebsiella pneumoniae* (hvKP), infection caused by CR-hvKP is more fatal (9). Its high virulence and carbapenem-resistant phenotype may lead to severe infections that are difficult to treat with current antibiotics. It is alarming that, despite the development of a new drug ceftazidime–avibactam, CR-KP resistant to ceftazidime–avibactam has begun to emerge in clinical practice, and its detection rate is increasing (10, 11). This poses a great threat to human public health.

Eravacycline is the world’s first fluorocycline antibacterial drug. As a broad-spectrum antibacterial drug, it inhibits protein synthesis by binding to the 30S small subunit of the bacterial ribosome, exhibiting potent antibacterial activity against carbapenem-resistant gram-negative bacteria, including *Klebsiella pneumoniae* (12, 13). Eravacycline was approved by the FDA in 2018 for the treatment of complex intra-abdominal infections. However, studies have indicated that it is also effective in other sites of infection, such as the lungs, skeletal muscle, and diabetic foot. Consequently, other clinical uses of this new antibacterial drug, including the treatment of bacterial pneumonia, are being gradually explored (14, 15). It has been reported that eravacycline exhibits high tissue penetration, with an effective concentration that is very high in most tissues, particularly in lung tissue. In a phase 1 study assessing the pharmacokinetics in healthy adults receiving intravenous eravacycline, the concentration of eravacycline in alveolar epithelial lining fluid (ELF) of these volunteers was significantly higher than those in plasma and alveolar macrophages (16).

To investigate the *in vivo* antibacterial activity of eravacycline on CR-hvKP, we screened three CR-hvKP strains from previously preserved isolates obtained from clinical patients (17). We analyzed their resistance genes, virulence genes, and virulence levels. Then, CR-hvKP was injected into the lung tissue of mice via endotracheal intubation to induce pneumonia. The therapeutic effect of eravacycline on pneumonia caused by CR-hvKP was investigated by studying the changes in body weight, the number of bacteria in each organ, the pathological damage of lung tissue, and the expression level of cytokines after eravacycline treatment, to provide a new theoretical basis for the clinical use of eravacycline in the treatment of CR-hvKP infection.

## MATERIALS AND METHODS

### Study samples

The experimental strains were isolated from clinical patients in Affiliated Chaohu Hospital of Anhui Medical University. Serum and neutrophils were extracted from the fresh blood of four healthy human volunteers after their verbal consent.

### Bacterial culture conditions

The preserved glycerol stock was retrieved from the −80°C freezer and streaked onto Luria-Bertani (LB) broth for isolation. Single colonies were obtained after overnight incubation at 37°C. A single colony was then ground and inoculated into LB broth, followed by shaking incubation at 37°C and 220 rpm for 16–18 h to obtain bacteria in the mid-logarithmic growth phase.

### Analysis of drug resistance and virulence genes

The resistance phenotypes, common resistance genes, and common virulence genes of clinical isolates KP78, KP98, and KP100 were determined in previous experiments (17). Three experimental strains were sent to Shanghai Major Bio-Pharmaceutical Technology Co., Ltd. for whole-genome sequencing to determine the prevalence of drug resistance and virulence genes.

### Biofilm

Biofilm formation was observed using scanning electron microscopy. A 0.5 McFarland bacterial suspension was prepared, and 200 μL of the bacterial suspension was added to a 24-well culture plate, supplemented with 2 mL of LB broth. After incubation at 37°C for 48 h, the LB liquid culture was removed and washed with PBS. The biofilm samples were then fixed with 2.5% glutaraldehyde fixative, and the samples were placed in different concentrations of ethanol (40%, 70%, 90%, and 99%) for 5 min. The samples were dried, sprayed with gold, and finally placed in the scanning electron microscope for observation.

### Serum killing assay

Experiments were performed as previously described in the literature (18). Serum from healthy adults was collected and stored at −80°C in a refrigerator. Approximately 10^6^ CFU of bacteria in the mid-phase of logarithmic growth were mixed with serum in a 1:3 ratio, incubated at 37°C for 0, 1, 2, and 3 h, and the number of viable bacteria was counted; each isolate was tested three times. *Klebsiella pneumoniae* ATCC700603 and ATCC43816 were used as negative and positive controls, respectively. The level of bacterial resistance to serum was graded according to the number of viable bacteria (19).

### *Galleria mellonella* larva infection assay

*Galleria mellonella* larvae are commonly used to assess the virulence of gram-negative bacteria (19). The specific experimental procedures were as follows. Thirty *Galleria mellonella* larvae weighing 250–350 mg were selected from each group. A bacterial suspension was generated from bacteria at the mid-phase of logarithmic growth, and 10 μL of bacterial suspension (1 × 10^4^ CFU) was injected through the left posterior second leg of the larvae. Infected larvae were placed in an incubator at 37°C, and survival was recorded every 24 h for 5 days. *Klebsiella pneumoniae* ATCC700603 and ATCC43816 were used as negative and positive controls, respectively.

### Neutrophil killing assay

Human neutrophils were isolated from the fresh blood of healthy adults. The layers of polymorphonuclear leukocytes and erythrocytes were separated by Ficoll density gradient centrifugation, and then polymorphonuclear leukocytes were successfully isolated by lysis of erythrocytes with sterilized water. Trypan blue staining was used to detect the viability of neutrophils, which were used immediately after isolation. Neutrophil killing assays were referenced in the literature, and some modifications were made (20). Freshly isolated neutrophils were suspended in RPMI 1640 medium, and bacteria in the mid-logarithmic growth phase were prepared as bacterial suspensions. The reaction system was divided into two parts: (i) 50 μL of neutrophils (1 × 10^5^ cells), 350 μL of RPMI 1640 medium, and 100 μL of bacterial suspension (4 × 10^6^ CFU); (ii) 400 μL of RPMI1640 medium, and 100 μL of bacterial suspension (4 × 10^6^ CFU). Following a 60 min incubation at 37°C, 500 μL of 0.5% Triton X-100 was added for 15 min to lyse the cells. Subsequently, a 10-fold gradient dilution of the stock solution using PBS buffer was inoculated into Mueller-Hinton Agar for counting the number of viable bacteria. The bacterial survival index was calculated by comparing the ratio of the two reaction systems, A and B. The experiment was repeated three times.

### Mouse model of pneumonia

Male C57BL/6J mice, aged 6–8 weeks (Gempharmatech Co., Ltd., Jiangsu, China), were used for acclimatization for 7 days before the experiment. During this period, the mice were fed a normal diet and water. The mice were then divided into three groups: the uninfected control group (PBS), the infection control group (KP78, KP98, and KP100), and the treatment group (KP78 + eravacycline, KP98 + eravacycline, and KP100 + eravacycline). On the day of the experiment, the mice were anesthetized by intraperitoneal injection of tribromoethyl alcohol (400 mg/kg, Nanjing Aibei Biotechnology Co., Ltd., Jiangsu, China). The uninfected control group received an injection of 50 μL of PBS into the lungs via endotracheal intubation, while the infection control group and the treatment group were injected with 50 μL of a bacterial suspension (1 × 10^7^ CFU/mL). Mice in the treatment group were injected with eravacycline (50 mg/bottle, Everest Medicines) via the tail vein. Regarding the dose of eravacycline, 10 mg/kg given intravenously every 12 h for three injections was chosen because we referred to the relevant literature (21, 22) and combined with the product instructions of eravacycline. The study comprised eight mice in each group. The status of the mice was examined during the infection process, and those who satisfied the criteria for euthanasia were sacrificed by cervical dislocation after anesthesia with tribromoethyl alcohol (400 mg/kg) in accordance with an authorized ethical protocol (IHM-AP-2024-008). Experimental mice in normal condition and those not meeting euthanasia criteria were euthanized at 48 h, and subsequent experiments were conducted using their corresponding tissues and organs.

### Number of bacteria in tissues and organs

Mice were killed 48 h after infection. The hearts, livers, spleens, lungs, and blood of mice were collected. A total of 100 μL of blood was plated on LB agar and incubated at 37°C, and the bacterial numbers were counted. After the organs were weighed, 900 μL of sterile PBS was added, and the tissue was ground into a homogenate. Then, a 10-fold gradient dilution of the tissue homogenate was performed, and 100 μL was plated on LB agar. The plate was incubated at 37°C, and bacterial numbers were counted.

### Cytokines were detected using flow cytometry

After the mice were anesthetized with isoflurane, serum was collected by retro-orbital bleeds. After the blood has naturally coagulated, it is centrifuged to obtain serum. The levels of IL-6, IL-1β, and MCP-1 in the serum of the mice were then detected using a mouse cytokine detection kit (Beadstar Biotechnology Co., Ltd., Hainan, China).

### Lung index

The intact lung tissue was removed, excess connective tissue was trimmed, and blood was washed away with PBS. After drying with sterile absorbent paper, the tissue was weighed. The lung index was calculated using the formula: lung weight (g)/mouse body weight (g) × 100%.

### Hematoxylin and eosin staining

A portion of the lung lobe from the mice was used as a pathological section, which was removed and then placed in 4% paraformaldehyde. After hematoxylin and eosin (H&E) staining, the degree of lung tissue injury was evaluated according to the following criteria (23): (i) alveolar congestion, (ii) hemorrhage, (iii) infiltration or aggregation of inflammatory cells in the alveolar cavity or blood vessel wall, and (iv) alveolar wall thickening and/or hyaline membrane formation. Four aspects were scored according to the severity of lesions (0 points: no lesions, 1 point: mild lesions, 2 points: moderate lesions, 3 points: severe lesions, and 4 points: very severe lesions), and the scores of each evaluation were added to the total pathological score. The slides of each group were numbered randomly and scored by a blinded pathology teacher with rich working experience. The total pathology scores were calculated by summing the individual scores.

### Reverse transcription-quantitative PCR

Total RNA was extracted from the lung tissue using the RNA Easy Fast Tissue/Cell kit (Tiangen, Beijing, China). The extracted RNA was reverse transcribed into cDNA using MightyScript Plus First Strand cDNA Synthesis Master Mix (Sangon Biotech, Shanghai, China), and then reverse transcription-quantitative PCR (RT-qPCR) was performed. The following primers were used: STAT1 F:5′-TACGGAAAAGCAAGCGTAATCT-3′ and R:5′-TGCACATGACTTGATCCTTCAC-3′. The relative expression levels of mRNA in different samples were analyzed by calculating 2^(−ΔΔCT)^ using β-actin (Sangon Biotech, Shanghai, China) as the normalization control.

### Western blot

The lung tissue homogenate was prepared with RIPA lysis buffer (P0013B, Beyotime Biotech Inc., Shanghai, China) containing protease and phosphatase inhibitors, and the resultant lysate was fully lysed and clarified using centrifugation. The protein concentration was determined using the bicinchoninic acid kits (Beyotime Biotech Inc, Shanghai, China). Protein samples were separated into 10% SDS-PAGE gels and transferred onto PVDF membranes. The membrane was blocked with TBST buffer (G2150, Servicebio Technology Co., Ltd. Wuhan, China) containing 5% bovine serum albumin and then incubated with primary antibody STAT1 (1:1,000, #9172T, Cell Signaling Technology, MA, USA), p-STAT1 (1:1,000, #7649T, Cell Signaling Technology, MA, USA), and β-actin (1:10,000, AC004, Abclonal, Wuhan, China) overnight at 4°C. Following this, the membranes were incubated with HRP-conjugated Goat anti-Rabbit IgG secondary antibody (1:8,000, AS014, ABclonal, Wuhan, China) at room temperature. Finally, the signals were obtained using the enhanced chemiluminescence kit (Beyotime, Shanghai, China) and observed using the Bio-Rad image analysis system (Bio-Rad, Hercules, CA, USA).

### Statistical analysis

GraphPad Prism 9.5.0 (GraphPad Software Inc., CA, USA) and Origin 2024 (OriginLab, MA, USA) were used for graphical presentation. SPSS Statistics 26.0 (IBM, NY, USA) was used for data analysis. Two groups of data, conforming to a normal distribution and homogeneous variance, were statistically analyzed using a *t*-test. The analysis was performed using the Mann–Whitney *U* test. Data comparisons among multiple groups were analyzed using one-way analysis of variance. The significance of the difference was indicated at *P*-values < 0.05.

## RESULTS

### Carriage of drug resistance genes and virulence genes in KP78, KP98, and KP100

**Fig 1:**
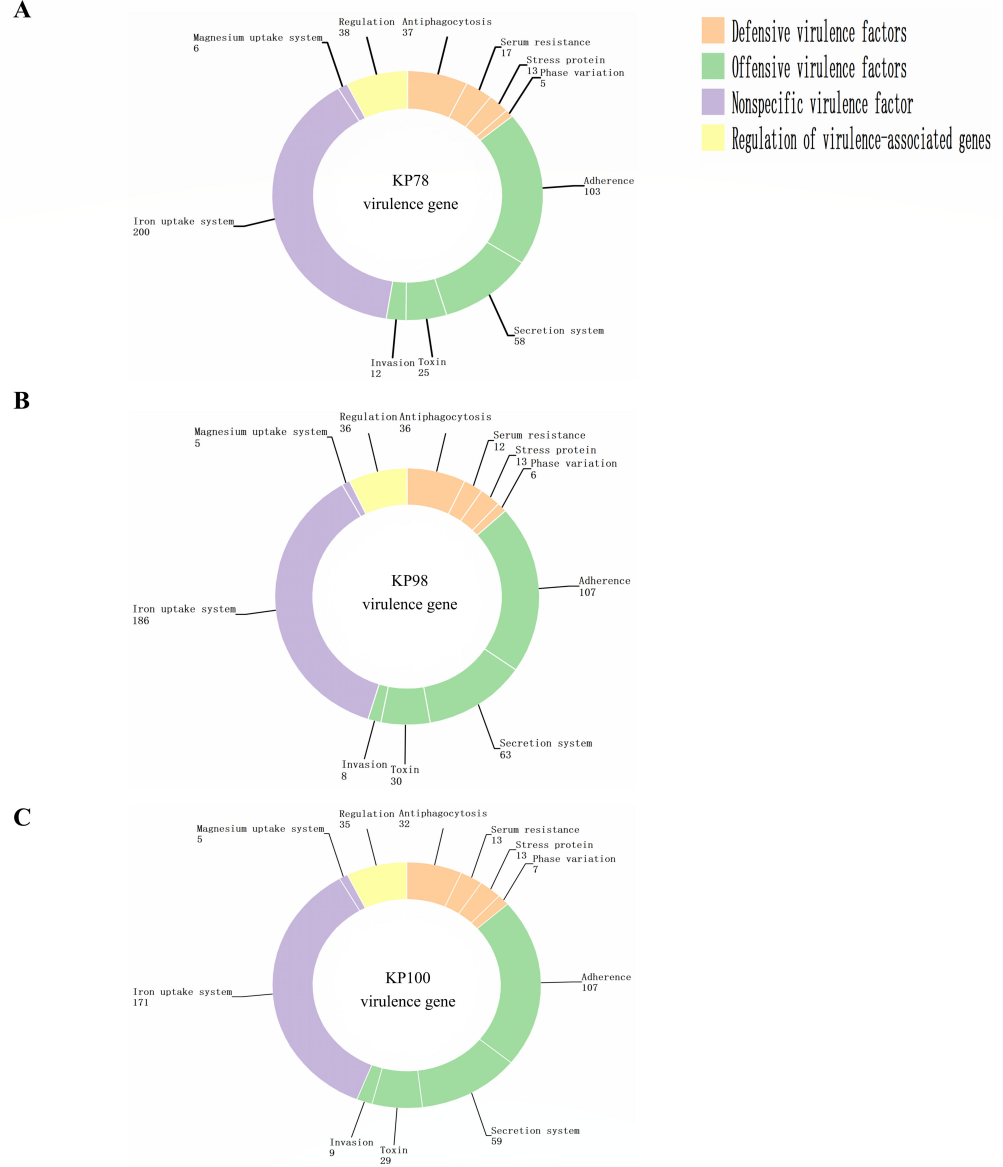
Distribution of virulence genes in three clinical isolates. (**A**) Distribution of virulence genes in isolates KP78. (**B**) Distribution of virulence genes in isolates KP98. (**C**) Distribution of virulence genes in isolates KP100.

In preliminary experiments, the drug resistance phenotypes, common resistance genes, and common virulence genes of the clinical isolates KP78, KP98, and KP100 were detected (17). The experimental strains harbored common virulence genes such as mrkD, fimH, rmpA, and ybts (Table 1). Most virulence genes carried by the strain are offensive virulence factors (such as adhesion and invasion) and iron transport systems (Fig. 1). Concurrently, the experimental strains exhibited resistance to clinically prevalent antibiotics, including carbapenems—the most common treatment for *Klebsiella pneumoniae* infections—such as imipenem, meropenem, and ertapenem. Notably, the experimental strains remained susceptible to the novel antibiotic eravacycline (Table 2).

**TABLE 1:** Common virulence genes carried by the experimental strain (17)

Strain	Virulence gene^*a*^					
	*rmpA*	*fimH*	*ybtS*	*mrkD*	*entB*	*kfu*
KP78	+	+	+	+	−	−
KP98	−	+	+	+	−	−
KP100	−	−	+	+	+	−

^*a*^ + indicates that the strain carries the corresponding virulence gene; − indicates that the strain does not carry that virulence gene.

**TABLE 2:** Antimicrobial resistance characteristics of the experimental strains (17)

Common antibiotic	MIC (μg/mL) for:		
	KP78	KP98	KP100
Eravacycline	≤0.12^*a*^	0.25^*a*^	≤0.12^*a*^
Meropenem	≥16^*c*^	8^*c*^	≥16^*c*^
Imipenem	≥16^*c*^	8^*c*^	≥16^*c*^
Ertapenem	≥8^*c*^	≥8^*c*^	≥8^*c*^
Ciprofloxacin	0.12^*a*^	≥4^*c*^	≤0.06^*a*^
Levofloxacin	≤0.12^*a*^	≥8^*c*^	0.25^*a*^
Cefepime	≥32^*c*^	16^*c*^	16^*c*^
Ceftazidime	32^*c*^	≥64^*c*^	16^*c*^
Ceftriaxone	32^*c*^	≥64^*c*^	8^*c*^
Cefotaxime	≥64^*c*^	≥64^*c*^	≥64^*c*^
Doxycycline	1^*a*^	4^*a*^	1^*a*^
Minocycline	2^*a*^	4^*a*^	2^*a*^
Chloramphenicol	4^*a*^	≥64^*c*^	4^*a*^
Colistin	2^*a*^	2^*a*^	≤0.5^*a*^
Cefoxitin	≤4^*a*^	16^*b*^	≤4^*a*^
Cefotetan	4^*a*^	4^*a*^	4^*a*^
Ceftazidime-avibactam	0.5^*a*^	1^*a*^	≤0.12^*a*^
Aztreonam	16^*c*^	≥64^*c*^	16^*c*^

^*a*^ The experimental strain is sensitive to this antibiotic.

^*b*^ Intermediate sensitivity.

^*c*^ Resistance.

### Biofilms of KP78, KP98, and KP100

**Fig 2:**
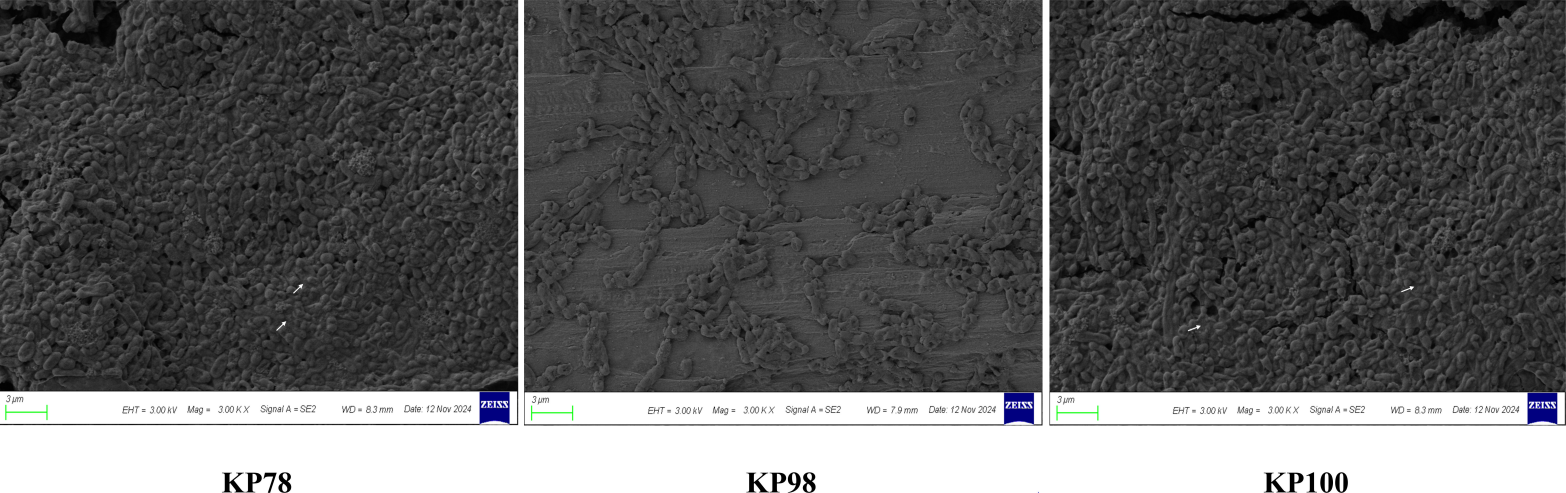
Biofilm formation status of the three experimental strains. The biofilm formation of KP78, KP98, and KP100 was analyzed using scanning electron microscopy, with the white arrows indicating the EPS.

In this study, we used a scanning electron microscope to examine the potential of KP78, KP98, and KP100 to form biofilms. As demonstrated in Fig. 2, KP78 and KP100 can form a biofilm. However, it was observed that only KP78 and KP100 of the three bacterial strains produced EPS and exhibited a mature biofilm structure. In contrast, KP98 demonstrated an inability to form a biofilm, exhibiting a limited number of bacteria attached to the carrier and an absence of EPS production.

### Virulence phenotypes of KP78, KP98, and KP100

**Fig 3:**
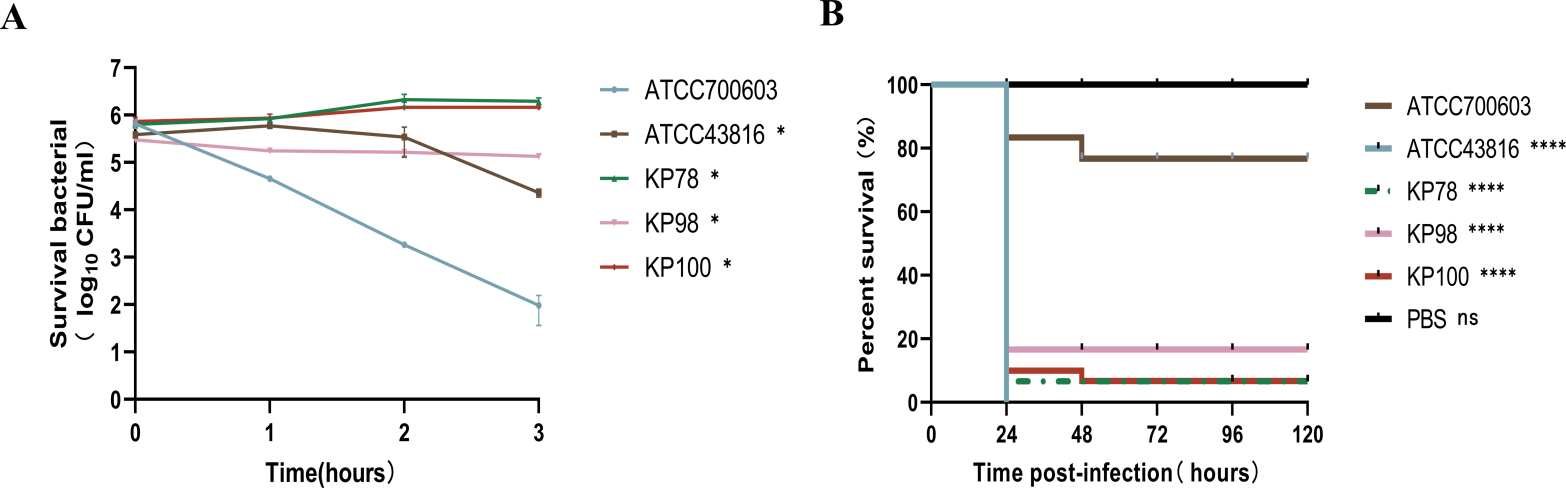
Virulence levels of the three clinical isolates. (**A**) Assessment of bacterial resistance to serum killing; ATCC43816 and ATCC700603 served as controls for high and low virulence. (**B**) Survival curves of *Galleria mellonella* larvae after infection; ATCC43816 and ATCC700603 were used as high and low virulence control groups, respectively, while PBS was used as the experimental control group. Data in panel A are shown as mean ± standard error of the mean. Asterisks represent comparisons between other groups and the low-virulence control strain ATCC 700603 (*P < 0.05 and ****P < 0.0001).

In the serum killing assay, the bacteria were graded according to the number of viable bacteria. ATCC700603 was used as a low virulence control (grade 1), which exhibited extreme sensitivity to serum. ATCC43816 was used as a highly virulent control with a grade of 3 and some serum resistance. The experimental strains KP78, KP98, and KP100 were assigned grades 6, 2, and 6, respectively. Experiments revealed that KP78 and KP100 can resist serum-mediated killing, with their bacterial counts even showing a slight increase over time. At the same time, KP98, despite not showing a trend of increasing bacterial numbers, demonstrated only a slight decrease in survival after co-culture with serum, indicating that it also possessed some degree of resistance to serum (*P* < 0.01; Fig. 3A). Figure 3B presents the survival curves of Galleria larvae injected with the experimental strain, PBS, the hypervirulent strain ATCC43816, and the hypervirulent strain ATCC700603 over a period of 5 days. It was evident that the larvae injected with PBS did not succumb to the treatment. In contrast, all larvae in the ATCC43816 group succumbed within 24 h. The survival rate of larvae infected with KP78, KP98, and KP100 was significantly lower than that of the ATCC700603 group, with a survival rate of less than 20% after 24 h (*P* < 0.0001; Fig. 3B). The experimental data demonstrated that the virulence levels of KP78, KP98, and KP100 were analogous to those of the high-virulence strain ATCC43816 and significantly higher than those of the low-virulence strain ATCC700603.

### KP78, KP98, and KP100 anti-phagocytic killing of human neutrophils

**Fig 4:**
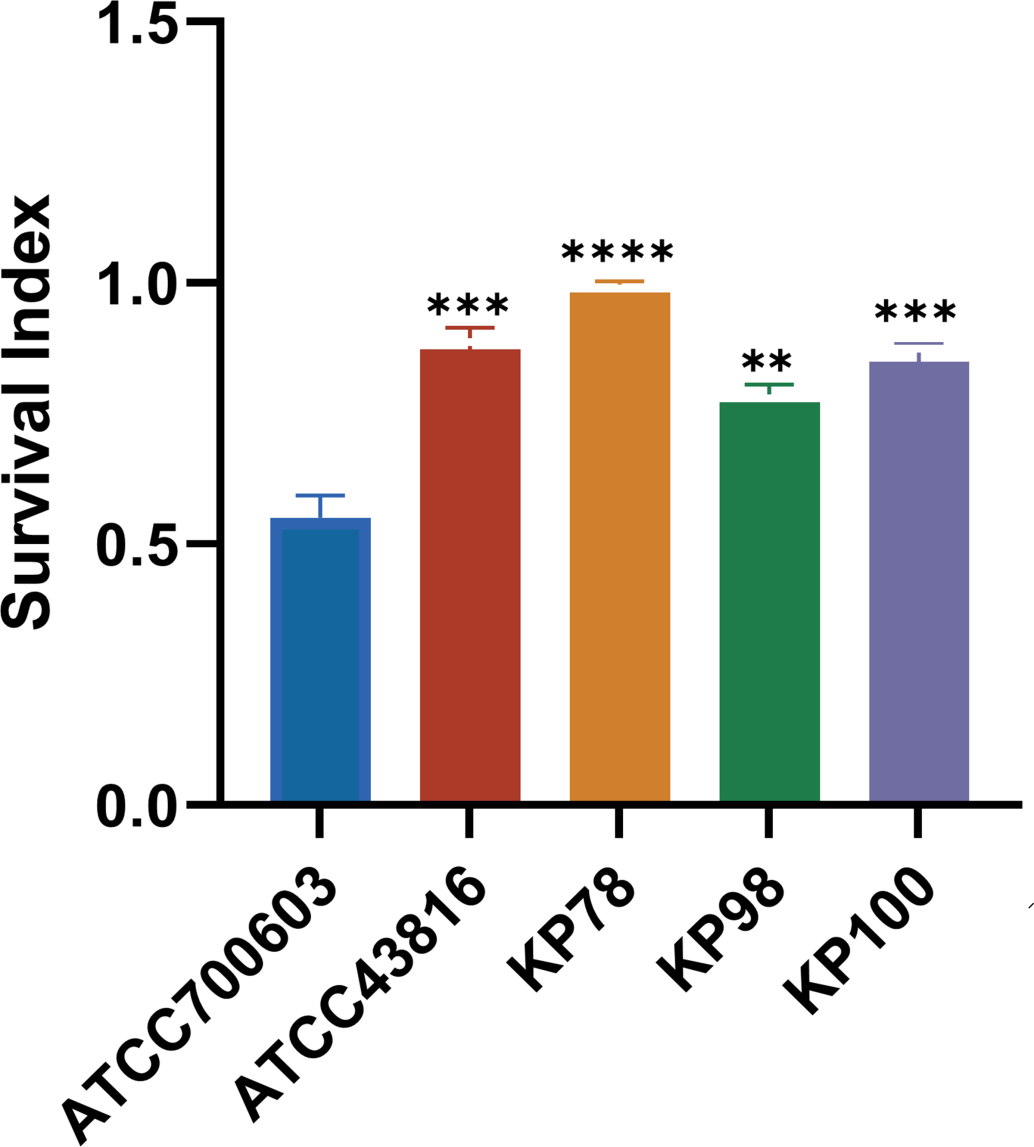
Resistance of three clinical isolates to neutrophil-mediated intracellular killing. ATCC43816 was used as a high positive control, and ATCC700603 was used as a negative control. Data are shown as mean ± standard error of the mean. Asterisks represent comparisons between other groups and the low-virulence control strain ATCC 700603 (**P < 0.01, ***P < 0.001, and ****P < 0.0001).

We tested KP78, KP98, and KP100 for resistance to neutrophil killing. Cell viability was assessed by Trypan blue staining and was over 95% for neutrophils isolated from the peripheral blood of healthy adults. Subsequently, the resistance of KP78, KP98, and KP100 to neutrophil-mediated intracellular killing was examined by assessing their survival index following co-culture with neutrophils. As demonstrated in Fig. 4B, the survival index of KP78, KP98, and KP100 is significantly higher than that of ATCC700603. This indicated that KP78, KP98, and KP100 exhibited robust resistance to neutrophil-mediated intracellular killing (*P* < 0.01; Fig. 4B).

### Weight change in mice and the bacterial load in each tissue and organ

**Fig 5:**
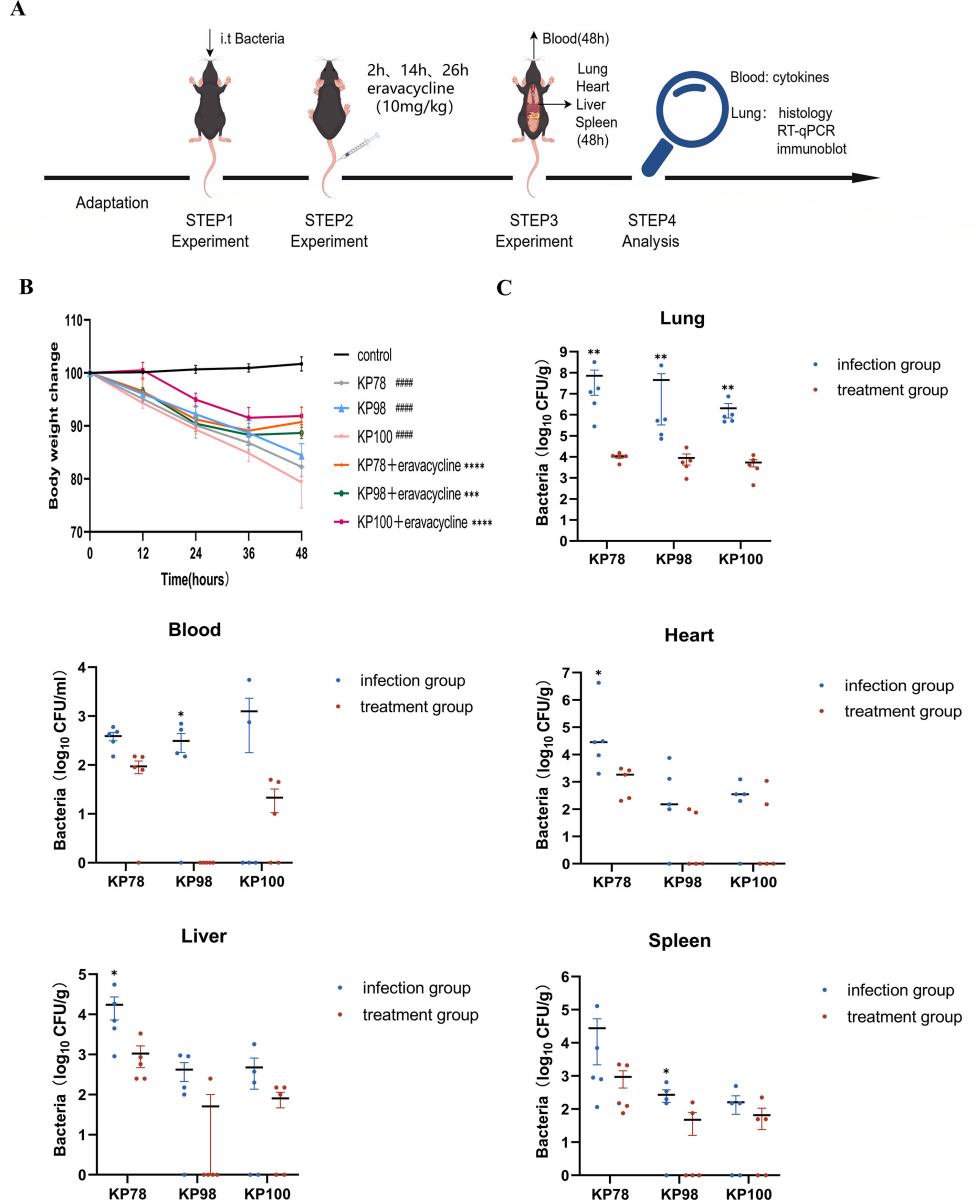
Changes in body weight and bacterial load in various tissues and organs of mice. (**A**) The experimental flowchart is presented. (**B**) Percentage of body weight of mice in the uninfected control, the infection control, and the treatment groups after 48 h. (**C**) Bacterial counts in the heart, liver, spleen, lung, and blood of mice in the infection control and the treatment groups. Data are shown as mean ± standard error of the mean, *n* = 5. Hashtags (#) represent the infection control group compared to the uninfected control group, and asterisks (*) represent the infection control group compared to the corresponding treatment group (*P < 0.05, **P < 0.01, ***P < 0.001, ****P < 0.0001, and ####*P* < 0.0001).

We infected the lungs of mice with CR-hvKP via tracheal intubation, and mice in the treatment group received eravacycline by tail vein injection after infection (Fig. 5A). Body weight changes were closely monitored before sacrifice in both the infection control group and the uninfected control group, each comprising five mice. Results showed that infected mice exhibited a continuous decline in body weight starting from infection, with a reduction of nearly 20% observed after 48 h (*P* < 0.0001, Fig. 5B). After 48 h, the surviving mice were killed to determine the number of bacteria in the heart, liver, spleen, lungs, and blood (Fig. 5C). The lung was the initial site of infection, and the number of bacteria in the lung tissue of the infection control group reached up to 10^8^ CFU. Furthermore, post-infection, KP78, KP98, and KP100 broke through the lung barrier and disseminated to other organs, including the heart, liver, and spleen, via the bloodstream. Following the injection of eravacycline, the rate of weight loss in the treatment group was observed to decelerate or even exhibit a slight increase (*P* < 0.001; Fig. 5B). Concurrently, a substantial decline in bacterial load was observed in the lung tissue of the treatment group (*P* < 0.01; Fig. 5C). A similar trend was noted in the heart, liver, spleen, and blood samples, where a decrease in bacterial counts was documented. These findings suggest that eravacycline exerts a substantial antibacterial effect on CR-hvKP.

### Pathological changes in the lung tissue of mice

**Fig 6:**
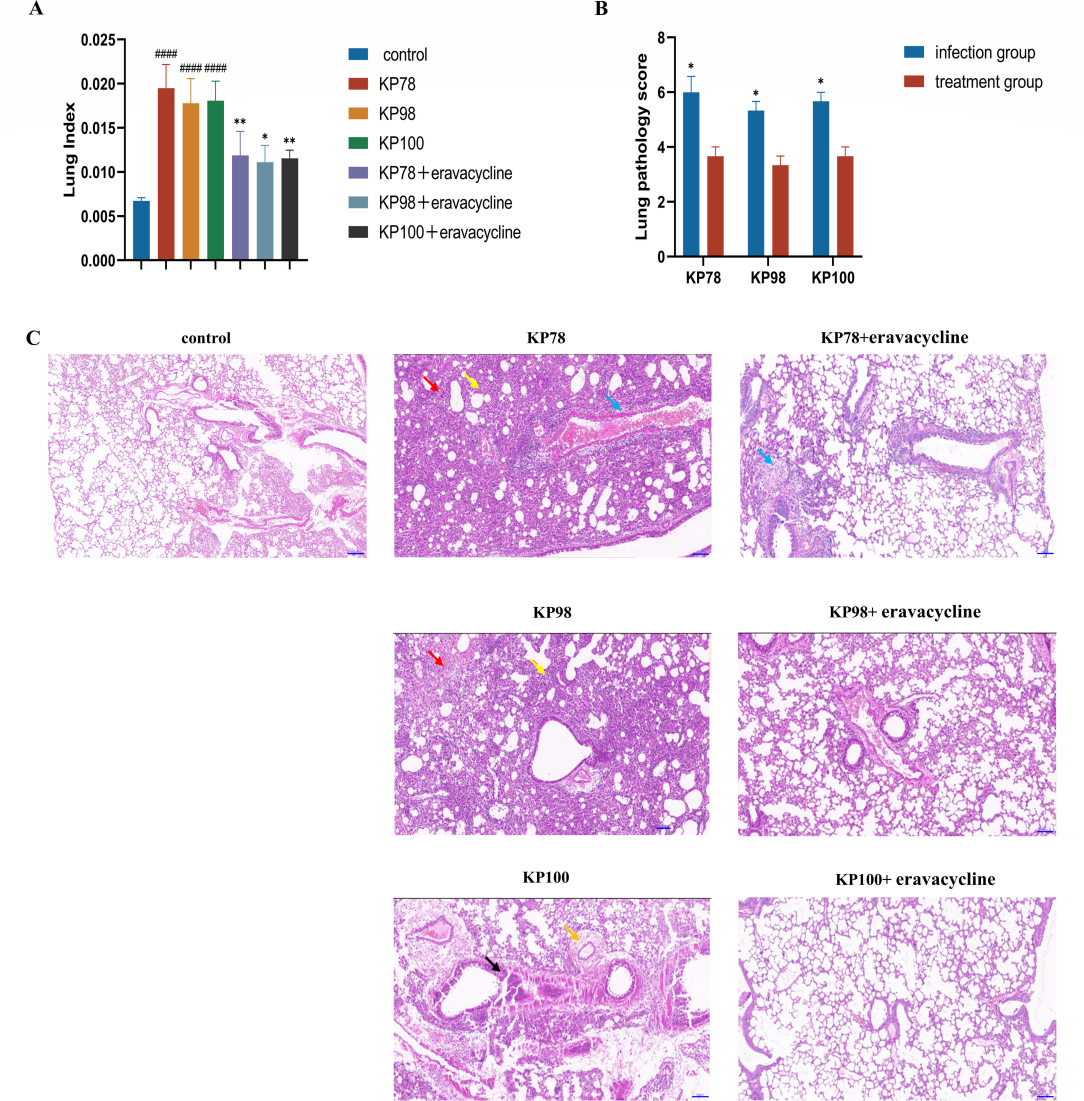
Degree of lung tissue lesions in each group of mice. (**A**) Lung index of mice in the uninfected control, the infection control, and the treatment groups. (**B**) The pathological scores of the lung tissues in the infection control and the treatment groups of mice. (**C**) Results of H&E staining of pathological sections of lung tissue of mice in each group. Inflammatory cell infiltration of the vessel wall (blue arrow), thickening of the alveolar wall (yellow arrow), hemorrhage (red arrow), bronchial epithelial cell necrosis/shedding (black arrow), and perivascular edema in the lung (orange arrow). The original magnification of the images was 100×. The bar in 100× is 100 µm. Data are shown as mean ± standard error of the mean, *n* = 3. Hashtags (#) represent the infection control group compared to the uninfected control group, and asterisks (*) represent the infection control group compared to the corresponding treatment group (*P < 0.05, **P < 0.01, and ####*P* < 0.0001).

Pulmonary index can reflect the severity of pulmonary edema to a certain extent. As demonstrated in Fig. 6A, the lung index of the infection control group was significantly higher than that of the uninfected control group (*P* < 0.0001; Fig. 6A). Although the lung index of the mice treated with eravacycline was still higher than that of the uninfected control group, it was significantly lower than that of the infection control group (*P* < 0.05; Fig. 6A). Histological analysis of the uninfected control group revealed the integrity of the bronchioles and alveolar cavities, as well as the normal thickness of the alveolar septum. In contrast, the infection control group exhibited alveolar damage, characterized by alveolar hemorrhage and inflammatory cell infiltration, as well as widened alveolar septa and compensatory alveolar expansion. Furthermore, the blood vessels in the alveolar wall exhibited dilatation and hyperemia, and the presence of necrotic cell fragments was observed in the bronchiolar cavity. However, treatment with eravacycline led to a substantial reduction in the severity of lung tissue lesions. The degree of lung tissue structural damage was reduced in the treatment group, with reduced infiltration of inflammatory cells and alleviated damage to the alveolar structure (Fig. 6C). Furthermore, a decline in lung pathological scores was observed in the treatment group compared to the infection control group (*P* < 0.05; Fig. 6B). These results indicated that eravacycline significantly attenuated the lung tissue injury caused by CR-hvKP infection.

### Changes in serum concentrations of cytokines IL-1β, IL-6, and MCP-1 in mice

**Fig 7:**
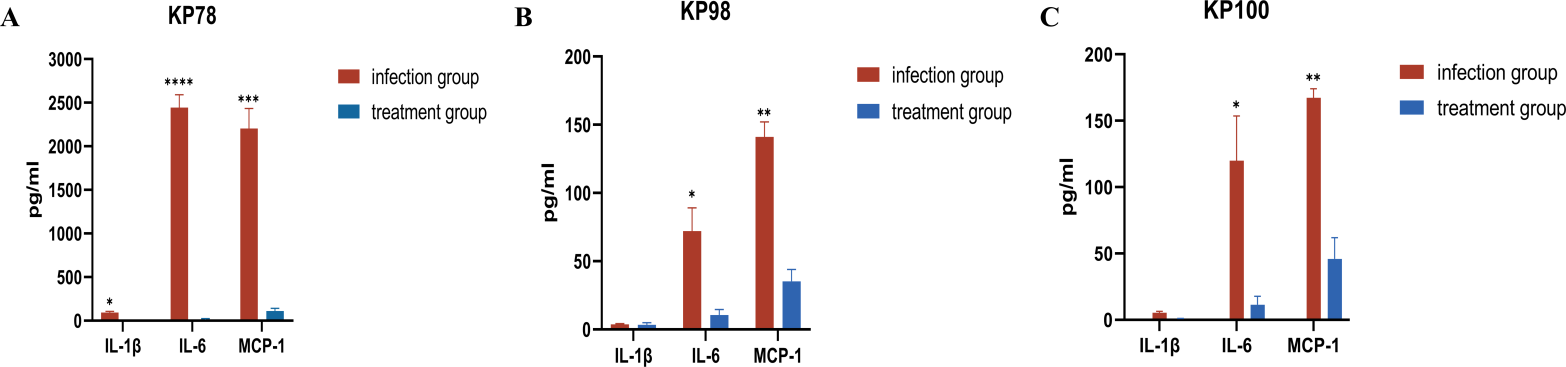
Serum cytokine concentrations in mice from each group. (**A**) Differences in serum levels of IL-1β, IL-6, and MCP-1 between the KP78-infected control and the treatment groups. (**B**) Differences in serum levels of IL-1β, IL-6, and MCP-1 between the KP98-infected control and the treatment groups. (**C**) Differences in serum levels of IL-1β, IL-6, and MCP-1 between the KP100-infected control and the treatment groups. Data are shown as mean ± standard error of the mean, *n* = 3. Asterisks represent the infection control group compared to the corresponding treatment group (*P < 0.05, **P < 0.01, ***P < 0.001, and ****P < 0.0001).

We examined the concentrations of the pro-inflammatory cytokines IL-1β, IL-6, and the chemokine MCP-1 in the serum of each mouse group. Mice infected with KP78, KP98, and KP100 exhibited heightened serum concentrations of IL-1β, IL-6, and MCP-1 in comparison to the uninfected control group (Fig. 7A and B). Among them, KP78 infection elicited the most substantial response, manifesting as a pronounced surge in serum concentrations of IL-6 and MCP-1. Although the elevation of cytokine concentrations after KP98 and KP100 infections was not as pronounced as in KP78 infections, the levels of IL-6 and MCP-1 were increased in comparison to uninfected mice. It was noteworthy that the levels of IL-1β, compared to those of IL-6 and MCP-1, increased exclusively in mice infected with KP78 (Fig. 7A). Next, we examined the concentrations of these cytokines in the serum of mice in the treatment group. The results demonstrated that the serum levels of IL-1β, IL-6, and MCP-1 in the mice were reduced to a certain extent after treatment with eravacycline (*P* < 0.05; Fig. 7B), suggesting that eravacycline treatment may alleviate the excessive activation of inflammatory factors caused by CR-hvKP pneumonia.

### Expression and activation of STAT1 in lung tissues of mice

**Fig 8:**
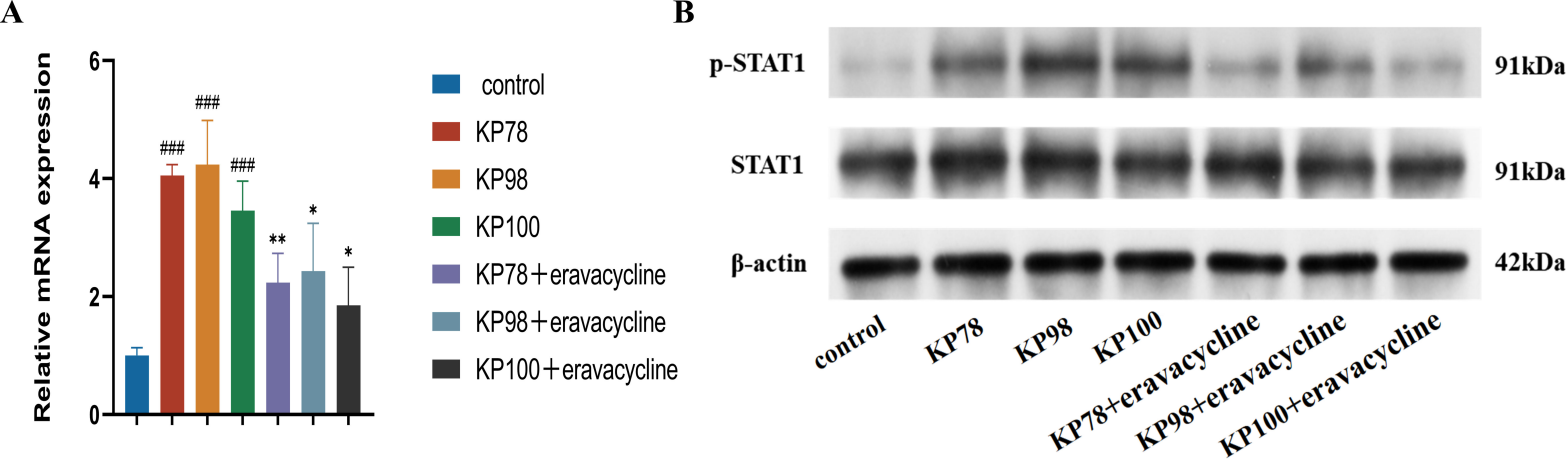
Expression and activation of STAT1 in mouse lung tissues. (**A**) mRNA expression levels of STAT1 in the lung tissues of mice across the uninfected control, the infection control, and the treatment groups. (**B**) Protein expression levels of p-STAT1 in the uninfected control, the infection control, and the treatment groups of mice. Data are shown as mean ± standard error of the mean, *n* = 3. Hashtags (#) represent the infection control group compared to the uninfected control group, and asterisks (*) represent the infection control group compared to the corresponding treatment group (*P < 0.05, **P < 0.01, and ###*P* < 0.001).

We detected the mRNA expression of STAT1 and the expression of p-STAT1 protein in mouse lung tissues using RT-qPCR and western blot. RT-qPCR results demonstrated that STAT1 mRNA expression levels in the lung tissues of the three CR-hvKP strains increased three- to fourfold following infection (*P* < 0.001; Fig. 8A). However, following treatment with eravacycline, STAT1 expression decreased (*P* < 0.05; Fig. 8A). Western blot analysis revealed that CR-hvKP infection led to an increase in p-STAT1 protein expression, which was suppressed in mice treated with eravacycline (Fig. 8B). These findings indicate that CR-hvKP infection enhances p-STAT1 expression and exacerbates lung inflammation. However, treatment with eravacycline has been shown to reduce STAT1 expression and inhibit p-STAT1 activation.

## DISCUSSION

*Klebsiella pneumoniae* is a common pathogen of both hospital-acquired and community-acquired pneumonia, and it is easily capable of causing bacteremic pneumonia. In previous reports, the bacteria detected in pneumonia patients infected with *Klebsiella pneumoniae* were predominantly CRKP and hvKP. However, in recent years, the detection rate of CR-hvKP has been increasing (24, 25). Common *Klebsiella pneumoniae* has caused considerable trouble in clinical treatment, and the emergence of CR-hvKP is undoubtedly worse. The high virulence of CR-hvKP causes more serious damage to infected patients, but the wide range of drug resistance makes the clinical treatment options very limited. Therefore, we should pay attention to CR-hvKP infection, and it is necessary to explore the efficacy of a new antibiotic, eravacycline, on CR-hvKP.

Our experimental strains exhibit resistance to several clinically common antibiotics (such as imipenem, meropenem, and ertapenem). More concerning is that KP78 and KP100 can also form biofilms, which may impair the bactericidal efficacy of eravacycline *in vivo*. Meanwhile, we also examined the virulence genes and virulence phenotypes of KP78, KP98, and KP100. It was found that all three strains carried multiple virulence genes. The virulence genes were found to be divided into four modules: defensive virulence factors, offensive virulence factors, non-specific virulence factors, and genes related to virulence regulation. These virulence genes confer different functions to the strains, such as anti-phagocytosis, serum resistance, adhesion, and invasion. It is worth noting that, as stated earlier, the experimental strains carry specific virulence genes (17). Typically, hvKP carries several virulence genes including mucus phenotype genes (rmpA); types 1 and 3 adhesins (fimH and mrkD); aerobactin, enterobactin (entB), and yersiniabactin (ybtS); the allantoin regulatory factor (allS); and iron uptake system (kfu) (26, 27). This is also a key characteristic distinguishing them from drug-resistant *Klebsiella pneumoniae*, endowing them with greater virulence. Our previous results showed that KP78 carried virulence genes mrkD, fimH, rmpA, and ybtS; KP98 carried virulence genes mrkD, fimH, and ybtS; KP100 carried virulence genes mrkD, fimH, and entB (17). Detection of virulence genes alone is insufficient, so we further validated the virulence level of the three experimental strains using the serum killing assays and the *Galleria mellonella* larval infection assay. The results showed that KP78, KP98, and KP100 all exhibited high virulence levels. When co-cultured with serum, the strains were able to resist the serum-mediated bactericidal effect to a certain extent, and even KP78 and KP100 showed a trend of increasing bacterial numbers within 3 h. Moreover, nearly all infected *Galleria mellonella* larvae died within 24 h. In conclusion, the carbapenem resistance of our test strains was accompanied by hypervirulence. There is no doubt that all three clinical isolates can be classified as CR-hvKP.

High virulence was observed in KP78, KP98, and KP100 in both the serum killing assay and the *Galleria mellonella* larval infection assay, and this high virulence phenotype was associated with greater lethality in these strains. Xu et al. observed significant neutrophil infiltration in hvKP-infected lung tissues, but not in CKP-infected lungs (28). hvKP often uses substances such as capsules to escape phagocytosis and killing by cells. When neutrophils cannot effectively eliminate bacteria, they will strengthen their recruitment (29, 30). Research has indicated that uncontrolled neutrophil activation may be a significant cause of disease deterioration during Kp infection. Resistance to phagocytic killing is not only a manifestation of the high virulence of hvKP but also a significant contributor to the cytokine storm caused by hvKP infection, ultimately leading to host death (31). Therefore, we also tested the resistance to neutrophil-mediated intracellular killing of the strains. The survival indices of KP78, KP98, and KP100 were all found to be high, thus indicating their capacity for resistance to neutrophil-mediated intracellular killing. What is more worrying is that KP78 and KP100 were capable of forming biofilms. Research has demonstrated that biofilms could disable the phagocytic killing mechanism of neutrophils and macrophages (32, 33). In instances where immune cells are unable to effectively eliminate bacteria, they will enhance their recruitment and the secretion of various factors, thereby inducing an excessive inflammatory response and causing damage to the host. In conclusion, after invading the lung tissue, most of KP78, KP98, and KP100 can resist clearance by pulmonary neutrophils, successfully colonize the lung, and spread to other organs, thereby triggering harmful inflammatory responses in the body.

Although eravacycline is currently approved for the treatment of complex intra-abdominal infections, there are further clinical values that also warrant investigation (14, 34). A study indicates that the drug concentration of eravacycline in the alveolar epithelial lining fluid (ELF) of healthy volunteers is significantly higher than the concentration in plasma (16). In theory, eravacycline could be used to treat pulmonary infections, but further studies are needed to confirm this. The present study explored this possibility. In fact, there is already a certain research foundation regarding the *in vivo* bactericidal efficacy of eravacycline (21, 35). Grossman et al. used *Streptococcus pneumoniae* to infect the lungs of mice and treated them with intravenous eravacycline at doses of 12 mg/kg, resulting in log10 CFU reductions in pulmonary bacterial burden of 3.9. In mice infected with *Pseudomonas aeruginosa*, the dose of 100 μg/mL of eravacycline also demonstrated significant antibacterial activity, with a marked reduction in bacterial load in the lungs. Undoubtedly, our experiments yielded similar results. The MIC of KP 78 and KP100 was ≤0.12, and the MIC of KP98 was 0.25; three test strains were susceptible to eravacycline. Furthermore, the antimicrobial activity of eravacycline against the three CR-hvKP strains was not limited to *in vitro* settings. Following tail vein injection of eravacycline, the body weight of infected mice began to stabilize after 36 h. The number of bacteria in the lung tissue decreased significantly, and the number of bacteria in other organs also showed a downward trend. It is noteworthy that eravacycline retains good antibacterial activity to alleviate the inflammatory damage caused by bacterial invasion despite the biofilm formation of KP78 and KP100 in CR-hvKP strains, which further suggests that eravacycline has therapeutic potential.

However, CR-hvKP infection causes not only bacterial colonization and dissemination, but also an excessive inflammatory response that is more deadly. Indeed, several studies have demonstrated that the virulence plasmid of hvKp plays a key role in mediating the infiltration of neutrophils and inducing the M1 polarization of macrophages, thereby causing a cytokine storm (28, 31, 36). Elevated IL-6 levels are a primary marker of cytokine storm, and their overexpression results in more severe bacteremia and higher mortality in KP-infected mice (37, 38). Furthermore, IL-6 can also affect the levels of other cytokines, such as enhancing MCP-1 production through trans-signaling pathways (39). MCP-1, a well-known chemokine, has been shown to recruit neutrophils to the lungs by upregulating CCR2 after KP infection and to induce macrophage migration and M1 polarization through the P38 MAPK signaling pathway (40, 41). Research has shown a positive correlation between the severity of pneumonia and the concentration of cytokines (42). The inhibition of the increase in cytokine concentrations, such as IL-6 and MCP-1, has been demonstrated to alleviate the inflammatory response caused by KP infection (23, 43, 44). In the present study, elevated serum concentrations of the pro-inflammatory cytokines IL-6 and MCP-1 were observed in infected mice. Treatment with the antibiotic drug, eravacycline, significantly reduced the serum concentrations of these pro-inflammatory cytokines. It is noteworthy that the increase in pro-inflammatory cytokine IL-1β can only be detected in the serum of mice infected with KP78. However, the increase in IL-1β is also inhibited by eravacycline. It was observed that eravacycline significantly alleviated the excessive inflammatory response caused by CR-hvKP infection by effectively killing CR-hvKP. The infiltration of macrophages and neutrophils in the lung tissue of the treatment group was significantly reduced, and the interstitial vascular dilatation and congestion were alleviated.

Notably, eravacycline can also reduce STAT1 expression levels by effectively eliminating CR-hvKP in lung tissue. Xu et al. demonstrated that STAT1 levels were significantly elevated on M1 macrophages and neutrophils in the lung tissue of CR-hvKP-infected mice, a key factor contributing to cytokine storms. Wu et al. significantly reduced the expression of MCP-1 and other cytokines by inhibiting the binding of STAT1 to the MCP-1 gene promoter, thereby alleviating LPS-induced pneumonia (45). Conversely, the loss of STAT1 has been observed to enhance bacterial clearance rates in murine models, with its activity demonstrating a close correlation with the exacerbation of pneumonia (46). In summary, the excessive inflammatory response following bacterial infection is closely associated with the activation of STAT1. In this study, we also observed a significant increase in both STAT1 mRNA levels and p-STAT1 expression in the lung tissues of infected mice. Following treatment with eravacycline, a significant decrease in STAT1 mRNA expression levels was observed, along with concomitant inhibition of p-STAT1 expression. This suggests that eravacycline reduces STAT1 expression levels and P-STAT1 activation by killing CR-hvKP cells in lung tissue, thereby mitigating injury to some extent.

In conclusion, our results indicate that eravacycline exhibits a potent antibacterial effect against CR-hvKP, which can effectively eliminate bacteria from pulmonary infections and inhibit the spread of bacteria. In addition, eravacycline significantly reduced the serum levels of IL-6, IL-1β, and MCP-1 and alleviated the pathological damage to the lung tissue. In future studies, we aim to conduct a comprehensive investigation of the signaling mechanisms involved in the remission of CR-hvKP pneumonia by eravacycline, thereby providing further insights into the treatment of bacterial pneumonia.

## Data Availability

The data generated and/or analyzed during the current study are available from the corresponding author upon reasonable request. The sequencing data of the three clinical isolates involved in this study have been deposited in the National Center for Biotechnology Information (NCBI) Sequence Read Archive (SRA) with the BioProject accession number PRJNA1290837.

## SUPPLEMENTAL MATERIAL

Fig. S1 aac.01237-25-s0001.tif The original WB image.

ASM does not own the copyrights to Supplemental Material that may be linked to, or accessed through, an article. The authors have granted ASM a non-exclusive, world-wide license to publish the Supplemental Material files. Please contact the corresponding author directly for reuse.
