# Supplementary figures and images for: Therapeutic effect of eravacycline against carbapenem-resistant hypervirulent Klebsiella pneumoniae in mouse models
Source: Antimicrob Agents Chemother. 2026 Feb 23;70(4):e01237-25. doi: 10.1128/aac.01237-25 (PMC13041357; doi:10.1128/aac.01237-25)

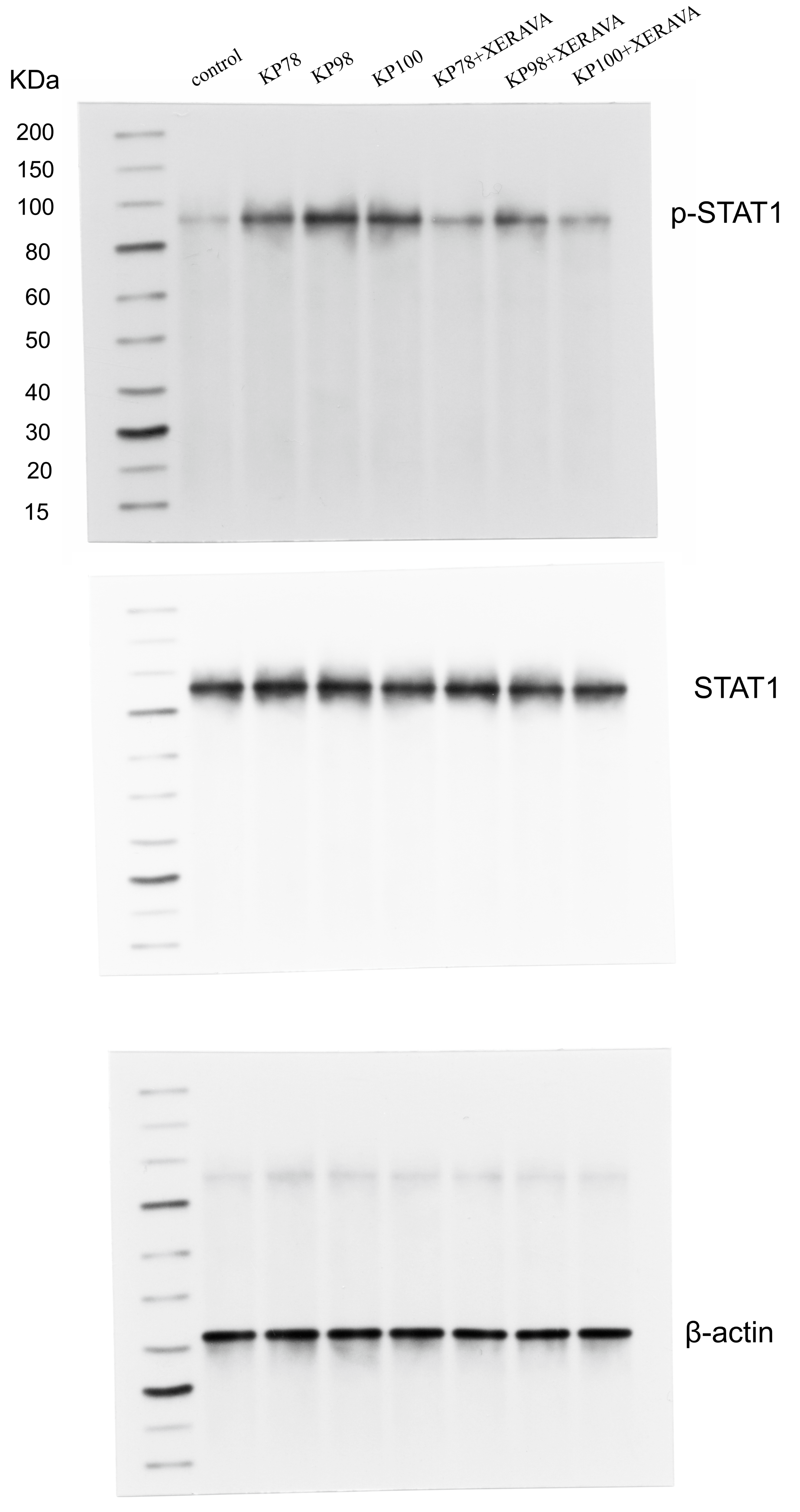

Supplement: Fig. S1 — The original WB image. [file aac.01237-25-s0001.tif]
